# To the Editors of the Medical and Physical Journal

**Published:** 1801-11

**Authors:** W. Wood

**Affiliations:** Manchester


### *To the Editors of the Medical and Phyfical Journal.*

GENTLEMEN,

AS the plate reprefenting the dimenfions of the pelvis of H. Rheubottom did not appear in the laft Journal, I fuppofe it will be in the next; and as you probably will have occafion to make fome mention of it, be fo good as to fay I omitted to ftate in the cafe, that the child was ftill born, but did not appear to have been dead for any great length of time, as no appearance of putrefaction had taken place, and the mother thought fhe had perceived it move brifkly about three or four hours before the operation.

I remain, with the greateft refpect,

Yours, &c.

*Manchefter, Oct.* 19, 1801. W. WOOD.

The Editors affure their readers, that whenever a plate is delayed, it is only for the fake of collecting a fufficient number of fubjects to fill an octavo page; and they will take care that no plate belonging to any volume fhall be delayed beyond the laft Number of that Volume.

---

